# Potency Biomarker Signature Genes from Multiparametric Osteogenesis Assays: Will cGMP Human Bone Marrow Mesenchymal Stromal Cells Make Bone?

**DOI:** 10.1371/journal.pone.0163629

**Published:** 2016-10-06

**Authors:** Alba Murgia, Elena Veronesi, Olivia Candini, Anna Caselli, Naomi D’souza, Valeria Rasini, Andrea Giorgini, Fabio Catani, Lorenzo Iughetti, Massimo Dominici, Jorge S. Burns

**Affiliations:** 1 Department of Medical and Surgical Sciences for Children & Adults, University Hospital of Modena and Reggio Emilia, Modena, Italia; 2 TPM, Science & Technology Park for Medicine, Mirandola, Modena, Italia; 3 CVBF - Consorzio per le Valutazioni Biologiche e Farmacologiche, Ospedale Pediatrico Giovanni XXIII, Bari, Italia; 4 Department of Orthopedic Surgery, University Hospital of Modena and Reggio Emilia, Modena, Italia; Instituto Butantan, BRAZIL

## Abstract

In skeletal regeneration approaches using human bone marrow derived mesenchymal stromal cells (hBM-MSC), functional evaluation before implantation has traditionally used biomarkers identified using fetal bovine serum-based osteogenic induction media and time courses of at least two weeks. However, emerging pre-clinical evidence indicates donor-dependent discrepancies between these *ex vivo* measurements and the ability to form bone, calling for improved tests. Therefore, we adopted a multiparametric approach aiming to generate an osteogenic potency assay with improved correlation. hBM-MSC populations from six donors, each expanded under clinical-grade (cGMP) conditions, showed heterogeneity for *ex vivo* growth response, mineralization and bone-forming ability in a murine xenograft assay. A subset of literature-based biomarker genes was reproducibly upregulated to a significant extent across all populations as cells responded to two different osteogenic induction media. These 12 biomarkers were also measurable in a one-week assay, befitting clinical cell expansion time frames and cGMP growth conditions. They were selected for further challenge using a combinatorial approach aimed at determining *ex vivo* and *in vivo* consistency. We identified five globally relevant osteogenic signature genes, notably TGF-ß1 pathway interactors; *ALPL*, *COL1A2*, *DCN*, *ELN* and *RUNX2*. Used in agglomerative cluster analysis, they correctly grouped the bone-forming cell populations as distinct. Although donor #6 cells were correlation slope outliers, they contrastingly formed bone without showing *ex vivo* mineralization. Mathematical expression level normalization of the most discrepantly upregulated signature gene *COL1A2*, sufficed to cluster donor #6 with the bone-forming classification. Moreover, attenuating factors causing genuine *COL1A2* gene down-regulation, restored *ex vivo* mineralization. This suggested that the signature gene had an osteogenically influential role; nonetheless no single biomarker was fully deterministic whereas all five signature genes together led to accurate cluster analysis. We show proof of principle for an osteogenic potency assay providing early characterization of primary cGMP-hBM-MSC cultures according to their donor-specific bone-forming potential.

## Introduction

Severe bone fractures often heal slowly with clinically challenging morbidity. Multipotent human Bone Marrow Mesenchymal Stromal Cells (hBM-MSC), frequently referred to as Mesenchymal Stem Cells, can be combined with biomaterial to help improve bone regeneration [1, 2]. A growing number of options are available for this approach, involving mesenchymal stem cells from different tissue sources [3], but concerns that alternative sources are not necessarily equivalent support choice of bone marrow derived hBM-MSC for bone therapy [4].

A discrepancy between the limited number of sourced autogenic hMSC to be found in the bone marrow and the number required for therapy, is nowadays resolved by expanding the cell population in culture according to current Good Manufacturing Practice (cGMP) [5]. To minimize risk of xenogenic immune incompatibility and prion infection, replacement of fetal bovine serum (FBS) with non-animal growth factors, e.g. human serum [6] or human platelet lysate (PL) [7, 8] is recommended.

Deteriorated cell function from the onset of senescence and concern for phenotypic drift mean that minimal timelines are recommended for cGMP production of hBM-MSC [9]. Though *ex vivo* expansion of primary hMSC populations obtained from the bone marrow is inherently finite [10–12], advances in culture methods allow cGMP facilities to grow 200 million stromal cells from a bone marrow sample within three weeks; a quantity considered sufficient for autologous therapy [13]. Nevertheless, beyond cell expansion limits, clinical outcomes can be thwarted by donor-specific heterogeneity in hBM-MSC functional potency [14].

A key prerequisite for hBM-MSC bone healing is retention of the specific potential to differentiate to osteoblasts rather than simply form stromal scar tissue [15]. Differentiating hBM-MSC mature to osteoblasts via a temporal cascade of selectively expressed regulatory transcription factors and osteogenic genes governing matrix deposition and mineralization [16]; such molecules and transition phenotypes may serve as readily detectable time-dependent osteogenic biomarkers [17]. Ideally, their measurement would provide indication of the status of a broad set of cellular parameters and bone forming competence. However, correlations between *ex vivo* expression of osteogenic biomarkers and bone formation *in vivo* have not been straightforward. Beyond early examples where only hBM-MSC strains with high levels of osteogenic markers *ex vivo* subsequently formed bone [18, 19], most studies over the past decade reveal surprisingly little direct correlation between bone forming potential and canonical biomarkers of *ex vivo* osteogenic differentiation, including mRNA expression levels of pro-collagen type I, alpha 1 (*COL1A1*), osteopontin (*SPP1*), alkaline phosphatase (*ALPL*) or runt related transcription factor 2 (*RUNX2*) [20–24].

Despite the above caveats, recent studies have aimed to correlate *ex vivo* measurements with bone formation, seeking more specifically informative indicators than proliferation [25]. Cell models that permitted genome-wide comparison of telomerized hMSC-TERT clones with different bone-forming ability, revealed that clone-specific bone-forming potential corresponded particularly well with the ex vivo gene expression of specific extracellular matrix proteins [26]. Notably, decorin (DCN), tetranectin (*CLEC3B*), collagen type-I, alpha 2 (*COL1A2*) and elastin (*ELN*) were bone-predictive genes induced by treatment of hMSC-TERT cells with osteogenic medium (OM) [27]. This agreed with prior views that the onset of differentiation was coupled to proliferation [28] and that the early rate of primarily type I collagen matrix production governed fracture healing [29].

However, the applicability of these correlative osteogenic biomarkers identified in telomerase-immortalized cells in FBS-based culture media to primary hBM-MSC grown in cGMP PL-based culture media has not been determined and prior gene expression assays have not necessarily conformed to the restricted time frames of preclinical cell expansion. Here, we developed a multiparametric phenotype-driven strategy to accommodate inter-donor heterogeneity whilst determining whether *ex vivo* osteogenic biomarker expression could indicate the subsequent bone-forming potential of cGMP-hBM-MSC from individual donors. Among donor-specific hBM-MSC populations that positively responded to OM with metabolic activation and matrix mineralization, we first verified expression of osteogenic biomarker genes in cGMP-hBM-MSC treated with OM containing FBS and then tested whether similar results were obtainable in OM containing PL (OM-PL). To be consistent with previous osteogenic biomarker studies, gene expression was first measured at comparable two-week time points. Then, to better match cGMP protocol timelines, we measured osteogenic biomarker expression after only one week of OM treatment.

We reasoned that cluster analysis seeking to correlate *ex vivo* gene expression with *in vivo* bone formation would need to be based on genes whose upregulation was statistically significant in all contexts. The bone-forming cGMP-hBM-MSC populations treated with OM-PL for one week shared seven upregulated osteogenic biomarker genes. Five of these genes were also consistently up regulated in cells positive for all our *ex vivo* tests of osteogenic differentiation. These five “signature genes” represented the varied cellular functions of matrix mineralization (*ALPL*), extracellular matrix synthesis (*COL1A2*, *DCN* and *ELN)* and transcriptional regulation (*RUNX2*). We here describe how using comparative cluster analysis the signature genes could promptly help discriminate heterogeneous donor-specific cGMP-hBM-MSC strains according to their bone-forming potential.

## Materials and Methods

### Cell Culture

cGMP facilities; Etablissement Français du Sang, Toulouse (France), Institute of Clinical Transfusion Medicine and Immunogenetics Ulm (Germany) provided donor-specific strains (n = 6, labelled #1-#6) of human bone marrow derived mesenchymal stem cells (hBM-MSCs) each population expandable to single clinical doses of at least 100 x10^6^ cGMP-hBM-MSC. The two-step protocol for unprocessed bone marrow cells involved seeding at an initial density of 50,000 white blood cells/cm^2^ in 300 mL complete medium in CellStack^™^ (Corning, Belgium) tissue culture vessels using PL based, animal-serum free alphaMEM medium (Lonza, Gaithersburg USA) [30]. Informed written consent from all six donors conformed to the Declaration of Helsinki and project approval by local ethical committees included testing of BM donors according to blood product guidelines.

Single passage cGMP-hBM-MSCs were shipped in frozen vials and thawed cells were seeded at 6 x10^3^ cells/cm^2^ in T75 flasks (Greiner Bio-one, Germany) incubated at 37°C with 5% humidified CO_2_ using maintenance medium (MM) consisting of Minimum Essential Medium (MEM) Alpha without nucleosides (Gibco^®^ Invitrogen, UK), supplemented with 8% (v/v) human Platelet lysate (PL), [31] 1% (v/v) L-Glutamine (Gibco^®^ Invitrogen, Belgium), 1 UI/mL heparin (Sigma-Aldrich, USA) and 10 μg/mL ciprofloxacin (HIKMA, Portugal). The cGMP-hBM-MSCs were replenished with fresh MM twice weekly and at 80–85% confluence were detached using trypsin 0.05%/EDTA 0.02% (PAA Laboratories, Austria) or TrypLE (Gibco^®^ Invitrogen, Belgium). The cGMP-hBM-MSCs were immunophenotypically and functionally characterized in the cGMP facilities ensuring high viability before shipping (Table 1).

**Table 1:** Characterisation of donor-specific cGMP-hBM-MSC population.

Donor	Gender	Age	CFU-F	CD34+	CD45+	CD73+	CD90+	CD105+	HLA-DR, DP, DQ+
#1	M	54	196	0.02	1.94	99.90	96.9	99.72	1.28
#2	F	51	30	ND	0.97	100	100	ND	2.9
#3	F	24	550	0.10	0.14	97.43	99.81	98.5	2.58
#4	M	42	68	0.00	4.4	100	100	99.76	0.4
#5	F	24	92	0.17	0.07	98.82	99.69	99.56	2.85
#6	M	21	98	0.03	0.05	99.9	100	93.14	0.28

*The number of Colony forming Unit-Fibroblast per 10^6^ mononuclear cells scored after the initial plating of the bone marrow sample. The % immunophenotype positivity was determined at passage 1 before shipment. CD34, Hematopoietic Progenitor Cell Antigen CD34; CD45, leukocyte common antigen; CD73, Ecto-5'-nucleotidase; CD90, Thymocyte antigen 1; CD105, Endoglin; HLA-DR, DP, DQ, Human leukocyte antigens. ND: not determined.

### Induction of *ex-vivo* osteogenic differentiation

Cells were seeded concurrently at a density of 10^4^/cm^2^ in 24-well multiwell plates (Greiner Bio-one) for Alizarin Red and Von Kossa staining and in T25 flasks (Greiner Bio-one) for RNA extraction. All culture vessels were incubated at 37°C with 5% CO_2_ in a humidified incubator (Thermo Scientific, Italy). At 85–90% cell confluence (≈3 days post seeding), we induced osteogenic differentiation. Two alternative differentiation protocol time courses were compared. Firstly, a previously established two-week (2W) protocol whereby cells were treated for the first week using osteogenic medium (OM) containing the inducing agents 10mM β-Glycerophosphate (ß-GP) (Sigma-Aldrich), 0.1 mM ascorbic acid-2-phosphate (AA) (Sigma-Aldrich), 10 nM Dexamethasone (Dex) (Sigma-Aldrich) supplemented with either either 10% (v/v) defined FBS (OM-FBS) or 8% (v/v) PL (OM-PL). For the second week, the OM-FBS or OM-PL was additionally supplemented with 100 ng/ml rhBMP-2 (Peprotech, UK). A two-week protocol facilitated biomarker data comparison with previous literature [26, 27, 32] and provided a more comprehensive comparison of FBS versus PL based OM. Secondly, to create an assay protocol more consistent with preclinical cell expansion timelines, we explored a one-week (1W) cell treatment protocol, whereby OM-FBS or OM-PL was supplemented with all the above inducing agents, including rhBMP-2 from the outset.

### Matrix mineralization

After OM treatment for one or two weeks, the extent of hBM-MSC matrix mineralization was characterised by Alizarin red S (ALZ) and Von Kossa (VK) staining. For the former, cells were washed at room temperature (RT) in PBS (1X), fixed in ice-cold methanol (100% v/v), washed with distilled water and stained with Alizarin Red S (Sigma) (1.5% v/v, pH 4.2) for 5 minutes to detect calcium precipitation. Stained monolayers were visualized by brightfield 10X magnification with an inverted microscope (Zeiss). For stain quantification, the plates were incubated at room temperature (RT) in the dark for 15 minutes with 500 μl of Cetylpyridinium Chloride (CPC) added to each well. The eluted dye solution was transferred to a fresh microcentrifuge tube, diluted ten times with PBS (1X) and dispensed as triplicate aliquots into a transparent 96-well plate (200 μL/well). Each measurement was performed at 562 nm on an ELISA reader (GDV, Roma).

Von Kossa staining to visualize phosphate and carbonate anions [33] was performed two weeks after OM treatment. Cell monolayers were washed in PBS (1X) at RT for 5 minutes and fixed in ice-cold methanol (100% v/v) for 4 minutes. The cells were rinsed twice in distilled water and incubated with 0.8 μm-filtered 1% silver nitrate (Sigma) for 30 minutes under a UV lamp. Stained samples were washed twice in distilled water and visualized with 10X magnification using an inverted microscope (Zeiss). Dark positive VK stained areas were quantified as a percentage of the total area using Image J software (http://rsb.info.nih.gov/ij/).

### Cell cycle activation biomarker *MKI67*^+^ expression

A primer set (Table 2) amplifying a 129 bp sequence spanning exons 13 and 14 of the *MKI67* gene recognised the short and long isoform splice variants encoding the Ki-67 nuclear protein associated with cell cycle activation. RNA extraction and quantitative real time reverse-transcriptase polymerase chain reaction (qRT-PCR) analysis was performed as described below.

**Table 2:** Primer sequences for polymerase chain reactions.

Gene	Primer Sequence	Amplified Length
***ACTB***	5'-ACCTTCTACAATGAGCTGCG-3' (sense)	**148 bp**
	5'-CCTGGATAGCAACGTACATGG-3' (antisense)	
***ALPL***	5'-GATGTGGAGTATGAGAGTGACG-3' (sense)	**142 bp**
	5'-GGTCAAGGGTCA GGAGTTC-3' (antisense)	
***BGLAP***	5'-CAGCGAGGTAGTGAAGAGAC-3' (sense)	**144 bp**
	5'-TGAAAGCCGATGTGGTCAG-3' (antisense)	
***BGN***	5'-TGGAGAACAGTGGCTTTGAAC-3' (sense)	**134 bp**
	5'-GTTGTGGTCTAGGTGGAGTTC-3' (antisense)	
***CADM-1***	5'-CCAGCGGTATCTAGAAGTACAG-3' (sense)	**146 bp**
	5'-TCACCCAAGTTACCATCACAG-3' (antisense)	
***CLEC3B***	5'-TCCTCCTCTGCCTCTTCTC-3' (sense)	**136 bp**
	5'-GTGTCCAGACGGCTCTTG-3' (antisense)	
***COL1A1***	5'-CCCCTGGAAAGAATGGAGATG-3' (sense)	**148 bp**
	5'-TCCAAACCACTGAAACCTCTG-3' (antisense)	
***COL1A2***	5'-AGGACAAGAAACACGTCTGG-3' (sense)	**146 bp**
	5'-GGTGATGTTCTGAGAGGCATAG-3' (antisense)	
***DCN***	5'-AAAATGCCCAAAACTCTTCAGG-3' (sense)	**146 bp**
	5'-GCCCCATTTTCAATTCCTGAG-3' (antisense)	
***DLX5***	5'-CTTATGCCGACTATAGCTACGC-3' (sense)	**124 bp**
	5'-CCATTCACCATTCTCACCTCG-3' (antisense)	
***ELN***	5'-CCTGGCTTCGGATTGTCTC-3' (sense)	**148 bp**
	5'-CAAAGGGTTTACATTCTCCACC-3' (antisense)	
***MKI67***	5'-AAAAGAATTGAACCTGCGGAAG-3' (sense)	**129 bp**
	5'-AGTCTTATTTTGGCGTCTGGAG-3' (antisense)	
***MSX2***	5'-CGGTCAAGTCGGAAAATTCAG-3' (sense)	**149 bp**
	5'-GGATGTGGTAAAGGGCGTG-3' (antisense)	
***PTH1R***	5'-ATGCTCTTCAACTCCTTCCAG-3' (sense)	**126 bp**
	5'-CTTTCGCTTGAAGTCCAGTG-3' (antisense)	
***RUNX2***	5'-TTCACCTTGACCATAACCGTC-3' (sense)	**148 bp**
	5'-GGCGGTCAGAGAACAAACTAG-3' (antisense)	
***SFRP1***	5'-AAGTGTGACAAGTTCCCCG-3' (sense)	**127 bp**
	5'-TGGCCTCAGATTTCAACTCG-3' (antisense)	
***SPP1***	5'-CAGTGATTTGCTTTTGCCTCC-3' (sense)	**149 bp**
	5'-ATTCTGCTTCTGAGATGGGTC-3' (antisense)	
***SP7***	5'-GCCAGAAGCTGTGAAACCTC-3' (sense)	**141 bp**
	5'-GCTGCAAGCTCTCCATAACC-3' (antisense)	
***TAZ***	5'-CGAATTCCTGCGTTTCAAGTG-3' (sense)	**147 bp**
	5'-GTGATTTTCTGTCCAAAGCGG-3' (antisense)	

***ACTB***, Beta-actin; ***ALPL***, alkaline phosphatase; ***BGLAP***, osteocalcin; ***BGN***, biglycan; ***CADM-1***, Cell adhesion molecule 1; ***CLEC3B***, tetranectin; ***COL1A1***, collagen type I alpha1; ***COL1A2***, collagen type I alpha2; ***DCN***, decorin; ***DLX5***, distal-less homeo box 5; ***ELN***, Elastin; ***MKI67***, antigen identified by monoclonal antibody Ki-67; ***MSX2***, Msh homeobox 2; ***PTH1R***, Parathyroid hormone 1 receptor; ***RUNX2***, Runt-related transcription factor 2; ***SFRP1***, Secreted frizzled-related protein 1; ***SPP1***, Osteopontin; ***SP7***, Osterix; ***TAZ***, Tafazzin.

### RNA extraction and quantitative Real-Time PCR (qRT-PCR)

Total cellular RNA was isolated using a single-step method with TRIzol (Invitrogen) according to the manufacturer's instructions. First-strand complementary cDNA was synthesized from 1 μg of total RNA using a revertAid H minus first-strand cDNA synthesis kit (Fermentas) according to the manufacturer's instructions. The reaction was terminated by heating at 70°C for 5 minutes. The single strand cDNA was quantified by spectrophotometer (Beckman Coulter DU^®^ 730) so as to use 10 ng of cDNA in each Real-Time PCR well.

Quantitative real-time PCR was performed using the Applied Biosystems StepOne^™^ Real-Time PCR System and the Fast SYBR^®^ Green Master Mix reagent. The quantification of gene expression for each target gene and reference gene was performed in separate tubes. Forward and reverse primers were designed using IDT PrimerQuest^®^ (http://eu.idtdna.com/PrimerQuest/Home/Index) ensuring they spanned an intron sequence to be specific for mRNA rather than genomic DNA. The relative expression level of the target gene was normalized to that of the endogenous reference ß-actin (*ACTB*) gene and the 2^-ΔΔCt^ cycle threshold method was used to calculate the relative expression levels of the target genes defined by the primers (Table 2).

### *In-vivo* Xenograft Bone Formation (BF) in immunodeficient mice

With University of Modena and Reggio Emilia ethical committee approval, the bone forming potential of the cGMP-hBM-MSC from each donor was tested using four implantation sites shared between two 8-week old NOD.CB17-Prkdc^scid/J^ mice. Previously described methods were modified to maintain the same cell/scaffold ratio as proposed for a clinical trial. Briefly, animals were anaesthetized with 3.6% isofluorane and the dorsal skin was shaved and cleaned. Incisions of ≈1 cm in length were performed on upper and lower dorsal regions on the back of each mouse. Blunt dissection was used to form a 3 cm long pocket and the graft was implanted therein. A total of 1.6 x10^6^ cells were mixed with 40 mg (≈40 granules) of hydroxyapatite β tricalcium phosphate (HA-βTCP 20:10, 1-2mm, Biomatlante, Vigneux de Bretagne, France) and implanted subcutaneously into the upper and lower dorsal flank of each mouse. The control samples were implanted with 40 mg HA-βTCP scaffold granules alone and the incisions were closed with sutures of ethicon vicryl rapide 5–0 (Johnson & Johnson). Cell/scaffold xenografts and control samples recovered from sacrificed animals after six weeks were transferred to 4% neutral buffered formalin for two days. Two PBS washes were followed by decalcification for 2–3 weeks in buffered 15% EDTA (pH 7.4). The decalcified HA-ßTCP implants were embedded in paraffin and 3 μm sections were stained with Hematoxylin and Eosin (H&E) for assessment of bone tissue formation. The percentage of bone tissue formed per total area [34] was quantified by two independent observers estimating the percentage of pink stained osteoid tissue relative to the total amount of scaffold and diversely stained surrounding stroma. At least 45 fields of view were examined for each donor, quantifying bone within near-equivalent total areas of scaffold.

### Phenotype driven selection of osteogenic biomarker “signature genes”

Eighteen candidate biomarker genes selected from a literature search were first assayed to determine time-dependent expression in response to one or two week osteogenic induction protocols. Genes showing consistent and significant upregulation in response to OM treatment were then examined in subsets of phenotypically functional cGMP-hBM-MSC populations to determine the average extent of gene upregulation according to each phenotype; ALZ^+^, VK^+^, *MKI67*^+^ and BF^+^. Genes significantly upregulated in all phenotypic contexts were classified as globally relevant osteogenic “signature genes”.

### Functional interaction networks

The Search Tool for the Retrieval of Interacting Genes (STRING) database (v9.05) explored possible relationships between the osteogenic signature gene products and whether they might be collectively associated with a particular regulatory network [35]. To maintain strict relevance, the three most confidently predicted functional partners were chosen to identify associated signaling pathways.

### Agglomerative Cluster Analysis

To determine inter-donor similarity according to signature gene expression patterns in OM treated cGMP-hBM-MSC, open-source software Cluster 3.0 (C clustering Library 1.50) visualized using Java Treeview v1.1.6r2, was used for agglomerative cluster analysis of the mRNA expression data. The best-fit cluster algorithm for our continuous variables was determined by comparing different linkage methods and distance measures. The averaged metric of Pearson centroid linkage with uncentered correlation distance was compared to more specific Single linkage with Euclidian distance metrics. Sample means were set to zero since our gene expression values represented a fold-increase in mRNA expression above a zero reference state determined by the control samples.

Pearson centroid linkage calculated a vector point from an average of all the items contained within the cluster, minimizing the effect of outlier values. Uncentered correlation distance provided a metric for the strength of linear association between two variables calculated from the sample values and their standard deviations. Euclidian distance, a geometric distance in multidimensional space, was appropriate for continuous variables sharing the same scale and assimilated the gene expression data better by taking the magnitude of changes more into account. The single linkage nearest-neighbor method may have drawbacks if the data has many points that bridge between clusters, but for our purposes it advantageously emphasized dissimilarity.

To understand the significance with which cluster analysis could distinguish donor-specific cGMP-hBM-MSC populations according to their extent of bone formation, the correlation coefficient indicating the degree of similarity between donors was plotted against the average amount of bone (mean%) formed by the respective members of the correlation.

### Biomarker signature gene verification; a role in outlier phenotypes

Cluster analysis of cells from outlier donor #6 that formed bone despite no apparent *ex vivo* mineralization, highlighted unusually high type I collagen gene upregulation. We adopted methods from evidence that inhibition of TGF-ß1 signaling corrected abnormal *COL1A1*-to-*COL1A2* gene expression ratios and increased alizarin red staining [36] to explore whether similar intervention could influence expression of the signature gene *COL1A2* and restore the *ex vivo* mineralization function to donor#6 cells. Cells were plated at a seeding density of 10^4^/cm^2^ in 24-well multiwell plates (Greiner Bio-one) for Alizarin Red staining and in T25 flasks (Greiner Bio-one) for RNA extraction. All culture vessels were incubated at 37°C with 5% CO_2_ in a humidified incubator. During the three days needed for cells to reach 85–90% confluence before OM treatment, MM was supplemented with either 40 mU/mL interferon-gamma (IFN-γ), a cytokine antagonistic to TGF-ß1 mediated collagen synthesis [37, 38] or 2 μM SB-431542, an inhibitor of the TGF-ß1 receptor activin receptor-like kinase (ALK5), [39]. The cells were then treated with OM-PL to induce matrix mineralization, harvesting mRNA for *COL1A2* gene expression analysis after one week and staining for Alizarin red after two weeks in OM-PL as described above.

### Statistical analysis

For the Real Time PCR analysis a two-tailed paired t test was applied to analyse differentially expressed genes between the control and induced groups in the OM-FBS or OM-PL inductions. Genes with >3-fold upregulation and p values less than 0.05 were considered significant. Individual gene expression graphs show standard error mean (S.E.M.) bars as a measure of the uncertainty in estimate of the mean. Graphs showing gene expression averaged from several donors show the standard deviation (S.D.) as a measure of the variability between donors.

## Results

### OM-PL promoted prompt induction of cGMP-hBM-MSC matrix mineralization

**Fig 1:**
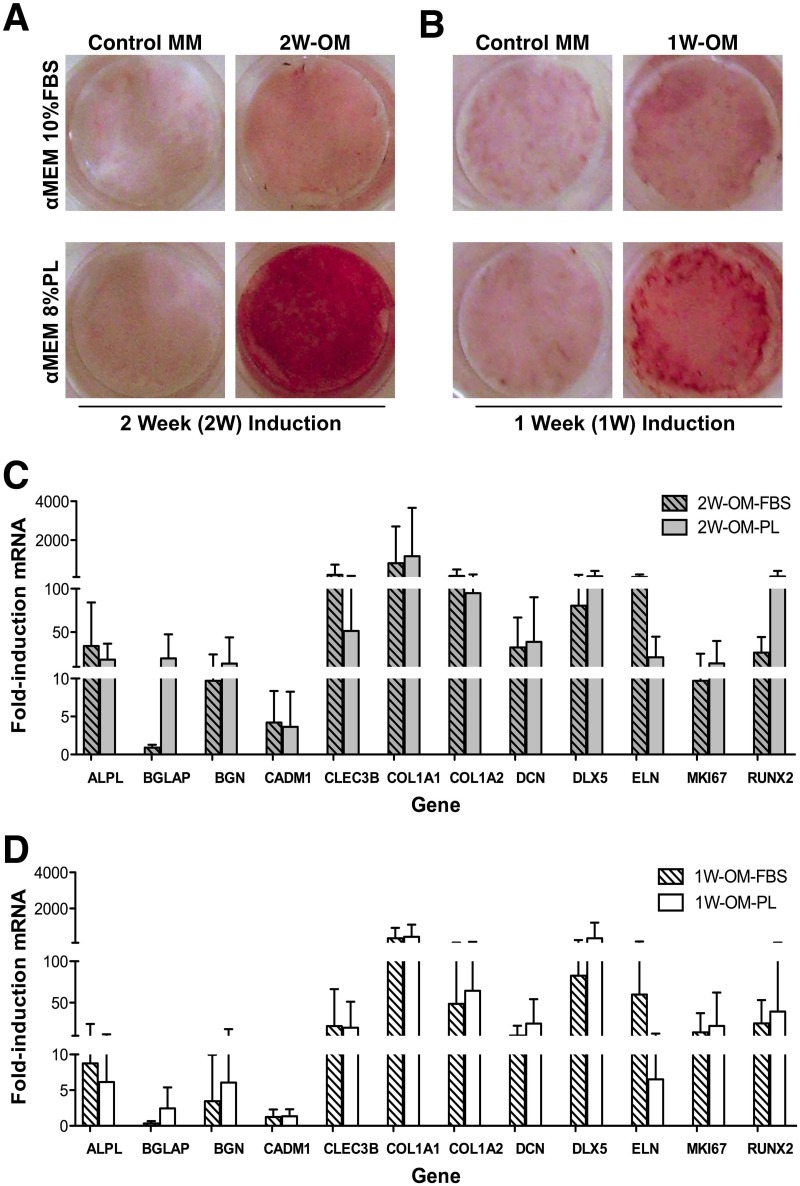
Osteogenic induction: Alizarin Red S staining and Osteogenic Biomarker expression. Representative photomicrographs of 24-well plate wells stained with Alizarin Red S after (A) two weeks or (B) one week of treatment with osteogenic medium (OM) based on Fetal Bovine Serum (FBS) or Platelet Lysate (PL). Parallel samples cultured in maintenance medium (MM) lacking osteogenic factors served as controls for spontaneous differentiation. Gene upregulation of 12 osteogenic biomarker genes in cGMP-hBM-MSC populations derived from six donors was measured after treatment with OM-FBS (hatched columns) or OM-PL (plain columns) for (C) two weeks or (D) one week. Measurements from triplicate determinations were all statistically significant (p < 0.05).

Pilot studies tested induction of differentiation in the six donor-specific cGMP-hBM-MSC populations, detecting matrix mineralization by ALZ staining after OM-PL or OM-FBS treatment. The low level ALZ staining of the control cells in MM (Fig 1A and 1B left panels) was increased when using OM-FBS and consistently more so using OM-PL for both 1W-OM and 2W-OM time points (Fig 1A and 1B lower right panels).

### OM-PL reproduced osteogenic biomarker gene induction seen with OM-FBS

Given positive differentiation outcomes from our protocols, we explored whether literature-mined osteogenic biomarker genes (often obtained with OM-FBS) were applicable to cells cultured with PL. We also needed to learn whether such biomarkers remained relevant for 1W-OM protocols. Of the 18 osteogenic biomarker genes initially selected, 6 genes; msh homeobox homologue 2 (*MSX2*), (parathyroid hormone receptor 1 (*PTH1R*), secreted frizzle-related protein 1 (*SFRP1*), osteopontin/secreted phosphoprotein 1 (*OPN/SPP1*), osterix (*SP7*) and tafazzin (*TAZ*) failed to show an appreciable fold-increase with respect to control samples in most of the six donor-specific cGMP-hBM-MSC populations (data not shown). In contrast, the remaining twelve osteogenic biomarker genes were robustly upregulated in cells treated with 2W-OM-FBS or 2W-OM-PL, in many cases over ten-fold (Fig 1C). Comparing OM-FBS with OM-PL the average level of gene upregulation was broadly equivalent, though exceptions included more upregulation of BGLAP (≈20X) and RUNX2 (≈5X) using OM-PL and more upregulation of ELN (≈5X) with OM-FBS. Nonetheless, more significant underlying donor-specific variation meant that most gene expression differences between OM-FBS and OM-PL were not statistically significant. After OM treatment for just one week, the osteogenic biomarker genes remained significantly measureable, despite an overall trend for lower levels of upregulation (Fig 1D), data in S1 Table. There remained a tendency for more upregulation of BGLAP (≈7X) using OM-PL and more upregulation of ELN (≈9X) using OM-FBS, but underlying donor heterogeneity again meant that these differences were not statistically significant. These osteogenic biomarker expression patterns confirmed cGMP-hBM-MSC differentiation in response to the 1W-OM-PL protocol.

### Osteogenic biomarker gene expression revealed inter-donor heterogeneity

**Fig 2:**
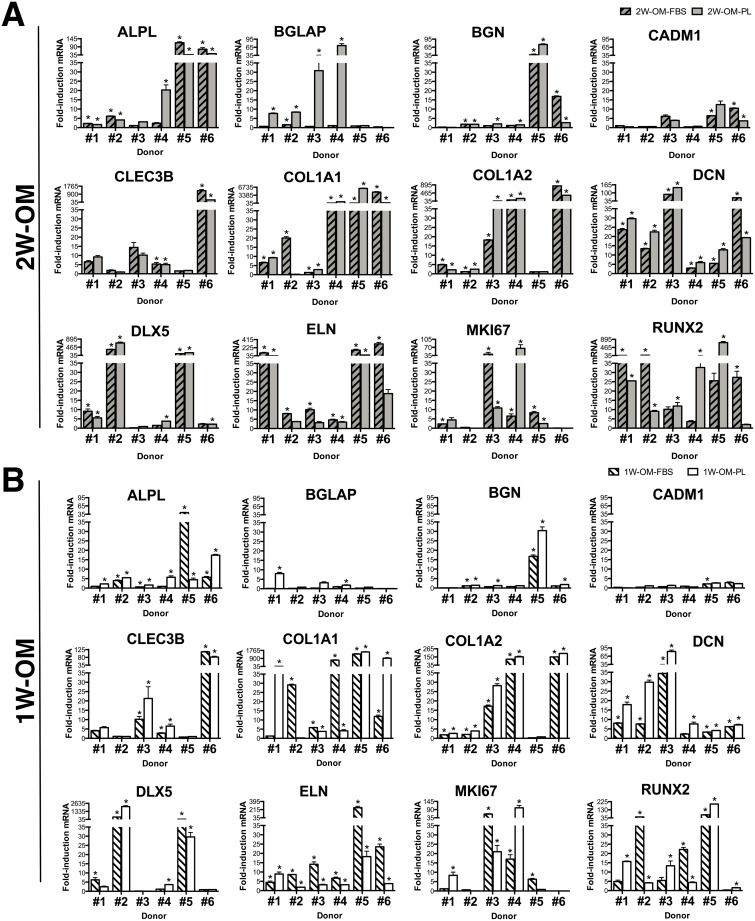
Inter-donor heterogeneity for osteogenic biomarker expression. The level of named gene upregulation in response to osteogenic medium treatment for (A) two weeks (2W-OM) or (B) one week (1W-OM) determined from triplicate measurements was tested to ensure statistical significance (*p < 0.05), error bars indicating standard error of the mean.

Individual cGMP-hBM-MSC populations (donors #1-#6) showed striking donor-specific gene expression patterns after OM treatment. Nonetheless, the two-week data (Fig 2A) showed close parity with data from the one-week differentiation protocol (Fig 2B), data in S1 Table. A comparison of geometric means indicated that most genes were upregulated to a greater extent in OM-PL than OM-FBS e.g. *BGLAP* (4.5x), *DCN* (2.15x), *BGN* (1.74x), *COL1A2* (1.63x), *ALPL* (1.62x), *CLEC3B* (1.37x), *RUNX2* (1.1x) and *DLX5* (1.05x). The most consistently upregulated genes (p <0.05) in OM-FBS or OM-PL were *ALPL*, *BGN*, *CLEC3B*, *COL1A2*, *DCN* and *DLX5* after two weeks and *BGN*, *CADM1*, *CLEC3B*, *COL1A2*, *DCN*, *DLX5* and *ELN* after one week of OM treatment (Table 3). One notable difference between use of FBS or PL was that in OM-PL *RUNX2* expression was more significantly covariant with other biomarker genes, namely *BGN*, *CADM1*, *COL1A1* and *ELN* at two weeks and *BGN*, *COL1A1* and *ELN* at one week (Table 4).

**Table 3:** Correlations for osteogenic biomarker gene upregulation in cGMP-hBM-MSC treated with OM-FBS or OM-PL for two weeks (2W) or one week (1W).

Gene	2W-OM-FBS versus 2W-OM-PL		1W-OM-FBS versus 1W-OM-PL	
	(r^2^)	p-value	(r^2^)	p-value
***ALPL***	**0.851**	0.032	-0.033	ns
*BGLAP*	0.186	ns	-0.084	ns
***BGN***	**0.911**	0.0116	**1.00**	6.81 E-5
***CADM1***	0.554	ns	**0.846**	0.034
***CLEC3B***	**1.00**	1.518 E-7	**0.987**	2.67 E-4
*COL1A1*	-0.185	ns	0.664	ns
***COL1A2***	**0.924**	0.008	**1.00**	3.55 E-7
***DCN***	**0.818**	0.047	**0.975**	0.001
***DLX5***	**0.995**	4.28 E-5	**0.991**	1.32 E-4
***ELN***	0.560	ns	**0.910**	0.012
*MKI67*	0.045	ns	0.244	ns
*RUNX2*	-0.026	ns	0.776	ns

(r^2^): coefficient of determination. ns: not significant

**Table 4:** Covariantly upregulated genes in cGMP-hBM-MSC treated with OM-FBS or OM-PL for two weeks (2W) or one week (1W).

Differentiation	Covariant Genes	Correlation	p-value
Protocol		(r^2^)	
**2W-OM-FBS**	***ALPL-BGN***	**0.987**	**2.50E-04**
	***BGLAP-DLX5***	**0.850**	**0.032**
	***CLEC3B-COL1A1***	**1.000**	**1.60E-07**
	***CLEC3B-COLIA2***	**0.994**	**4.90E-05**
	***COL1A1-COL1A2***	**0.995**	**3.80E-05**
**2W-OM-PL**	***BGLAP-MKI67***	**0.960**	**0.002**
	***BGN-CADM1***	**0.946**	**0.004**
	***BGN-COL1A1***	**0.995**	**3.40E-05**
	***BGN-ELN***	**0.832**	**0.040**
	***BGN-RUNX2***	**0.998**	**3.90E-06**
	***CADM1-COL1A1***	**0.927**	**0.008**
	***CADM1-RUNX2***	**0.932**	**0.007**
	***CLEC3B-COL1A2***	**0.878**	**0.021**
	***COL1A1-ELN***	**0.822**	**0.045**
	***COL1A1-RUNX2***	**0.996**	**2.60E-05**
	***ELN-RUNX2***	**0.846**	**0.034**
**1W-OM-FBS**	***ALPL-BGN***	**0.995**	**3.00E-05**
	***ALPL-COL1A1***	**0.840**	**0.037**
	***ALPL-ELN***	**0.995**	**4.00E-05**
	***BGN-COL1A1***	**0.868**	**0.025**
	***BGN-ELN***	**0.999**	**3.20E-06**
	***COL1A1-ELN***	**0.859**	**0.029**
	***DCN-MKI67***	**0.898**	**0.015**
**1W-OM-PL**	***ALPL-CLEC3B***	**0.881**	**0.020**
	***BGN-COL1A1***	**0.856**	**0.029**
	***BGN-ELN***	**0.902**	**0.014**
	***BGN-RUNX2***	**0.993**	**6.60E-05**
	***CADM1-COL1A1***	**0.869**	**0.025**
	***COL1A1-RUNX2***	**0.820**	**0.046**
	***ELN-RUNX2***	**0.936**	**0.006**

(r^2^): coefficient of determination.

Inter-donor heterogeneity included individual examples of high gene induction (Fig 2B); e.g. *BGN* was only upregulated markedly in cells from donor #1 (16.7-fold with OM-FBS, p = 0.00018 and 30.4-fold with OM-PL, p = 0.00014), similarly *DLX5* in cells from donor #2 (64-fold with OM-FBS, p = 0.0004 and 2106-fold with OM-PL, p = 0.0003) and *ELN* in cells from donor #5 using OM-FBS (258.3-fold, p = 0.03). In contrast, DCN showed significant OM-PL mediated gene induction in all donors, ranging from 3.8-fold, p = 0.0001 in cells from donor #5 to 82-fold, p = 0.0002 in cells from donor #3.

Focusing on the assay most relevant to the clinical protocol, 1W-OM-PL treatment, two-way analysis of variance indicated that the donor-specific influence on gene upregulation was significant (p<0.001) accounting for over 70% of the total variance in seven of the twelve osteogenic biomarker genes, namely *BGN*, *CADM1*, *CLEC3B COL1A1*, *COL1A2*, *DCN* and *RUNX2*. Overall, there was considerable variation in the extent of gene upregulation during differentiation and most importantly, osteogenic biomarker gene expression measured after only one week of OM-PL treatment was suitable for identification of inter-donor differences.

### Inter-donor heterogeneity for OM-PL-induced ALZ^+^, VK^+^, *MKI67*^+^ *ex-vivo* phenotypes

**Fig 3:**
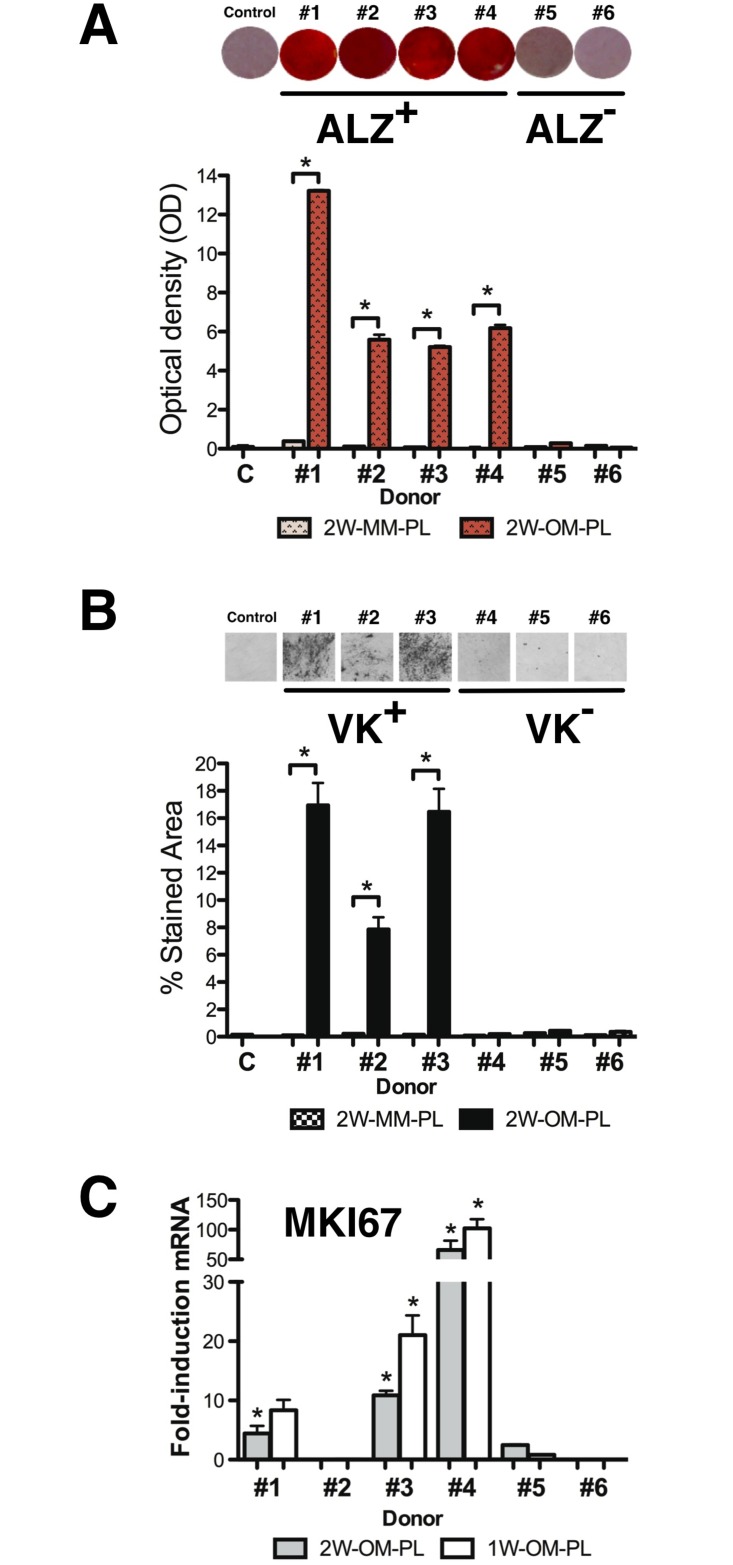
Inter-donor heterogeneity for OM-PL-induced ALZ^+^, VK^+^, *MKI67*^+^ *ex-vivo* phenotypes. After two weeks of osteogenic induction in OM-PL, the hBM-MSCs were stained for matrix mineralization. (A) Representative photomicrographs (10X) of donor-specific cGMP-hBM-MSC populations positive for Alizarin red S stain (ALZ+) and histogram of eluted dye staining intensity measurement at 562 nm (*p < 0.05). (B) Representative photomicrographs (10X) of donor-specific cGMP-hBM-MSC populations positive for Von Kossa stain (VK+) and histogram of positively stained area quantified using Image J software (*p < 0.05). (C) Histogram showing the extent of gene upregulation of *MKI67* determined after induction with OM-PL for two weeks (dark columns) or one week (light columns). Measurements from triplicate determinations that were statistically significant are indicated (*p < 0.05).

There was relatively little ALZ^+^ staining observed after OM treatment for one week, but after two weeks, two main donor-specific phenotypes were found. Cells from four donors (#1, #2, #3, #4) exhibited extensive matrix mineralization and were characterized as ALZ^+^ with OD values ranging between 5 and 13, significantly greater than the negative control samples (p = 0.0003) (Fig 3A). In contrast, cells derived from donors #5 and #6 exhibited weak ALZ^+^ staining, resembling that of negative control cells and were characterized as ALZ^-^ with a background level OD less than 0.5 (p<0.015).

Over the same two-week treatment period, only three of the four ALZ^+^ donors, namely #1, #2, #3 were also VK^+^, showing diffuse areas of calcium phosphate stained by black silver ion particles covering 8 to 16% of the surface area evaluated by Image J software (Fig 3B). In contrast, cells from donors #4, #5 and #6 had no significant VK staining and resembled the non-OM treated negative control cells, data in S2 Table.

Given proposals that proliferation plays an important role in the early stages of differentiation, we analyzed *MKI67* expression in OM-PL treated cells. The inter-donor heterogeneity for *MKI67* upregulation did not correspond directly with the mineralization phenotypes. After 2W-OM-PL treatment the low *MKI67* expression of donors #2, #5, #6 remained low, whereas donors #1, #3 and #4 showed 4 to 66-fold *MKI67* upregulation. After 1W-OM-PL treatment, the significant *MKI67* upregulation for donors #1, #3 and #4 ranged from 8 to 102-fold (p<0.05) (Fig 3C), data in S1 Table. Thus both time points confirmed inter-donor heterogeneity for *MKI67*^+^ expression with greater upregulation of the active cell cycle biomarker during early phases of osteogenic differentiation.

### Inter-donor heterogeneity for the extent of bone formation *in-vivo*

**Fig 4:**
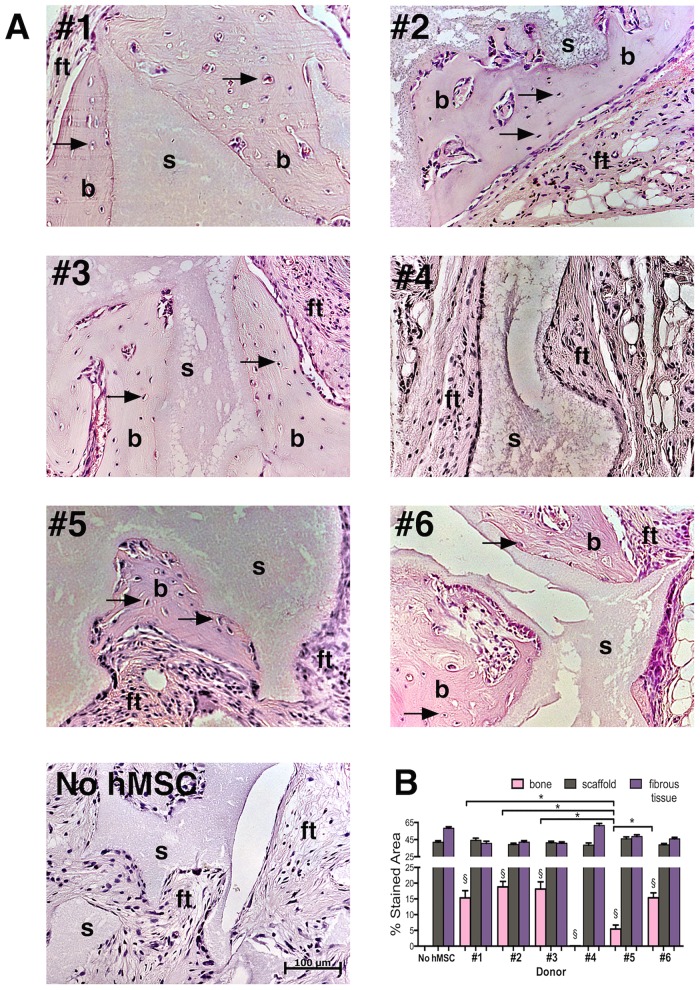
Inter-donor heterogeneity for bone formation *in-vivo*. Photomicrographs of H&E stained sections of decalcified paraffin-embedded Xenografts under bright field illumination. The Xenografts consisted of hydroxyapatite ß-tricalcium scaffold granules seeded with cGMP-hBM-MSC derived from (A) donors #1 to donor #6 respectively. Regions adjacent to the scaffold (s) contained newly formed osteoid bone (b) stained more homogeneously pink relative to the surrounding fibrous tissue (ft) and contained numerous osteocytes within lacunae (arrows). A representative section of the control implant of hydroxyapatite ß-tricalcium scaffold granules without cells revealed scaffold (s) and fibrous tissue (ft) only. (B) Histogram of the histological section area governed by scaffold (grey column), stromal fibrous tissue (purple column) or bone osteoid matrix (pink column) showing significant bone formation (§p<0.05). Donor heterogeneity with regard to the relative amount of bone formed showed statistically significant differences (*p < 0.05). Scale bar = 100 μm.

All NOD/SCID mice subjected to xenograft implants were healthy after the experimental procedures and the small implant incisions healed within 7 days without infection or complications. H&E stained sections of hBM-MSCs and HA-βTCP xenografts six weeks post-implantation revealed donor-specific heterogeneity for the extent of ectopic bone formation among fields of view capturing equivalent areas of scaffold. cGMP-hBM-MSC derived from donors #1, #2, #3 and #6 had a good positive bone-forming phenotype (BF^+^) forming new bone comprising 15–18% of the total tissue section area (Fig 4A and 4B). In contrast, xenografts specific to donor #4 showed cells aligned along the scaffold surface and vascularization of the tissue but no significant bone formation (Fig 4A). Unlike the other bone-forming xenografts, donor #5 cells failed to show any evidence for hematopoietic territories adjacent to the newly formed bone and the amount of bone formed by donor #5 cells (5.4%) was significantly less (p<0.05) than the average bone formation among the other bone-forming donors (16.91%) (Fig 4B), thus it was defined as poor bone-forming (BF^-^), data in S3 Table.

### Biomarker characterization of ALZ^+^, VK^+^, *MKI67*^+^, BF^+^ cell populations

**Fig 5:**
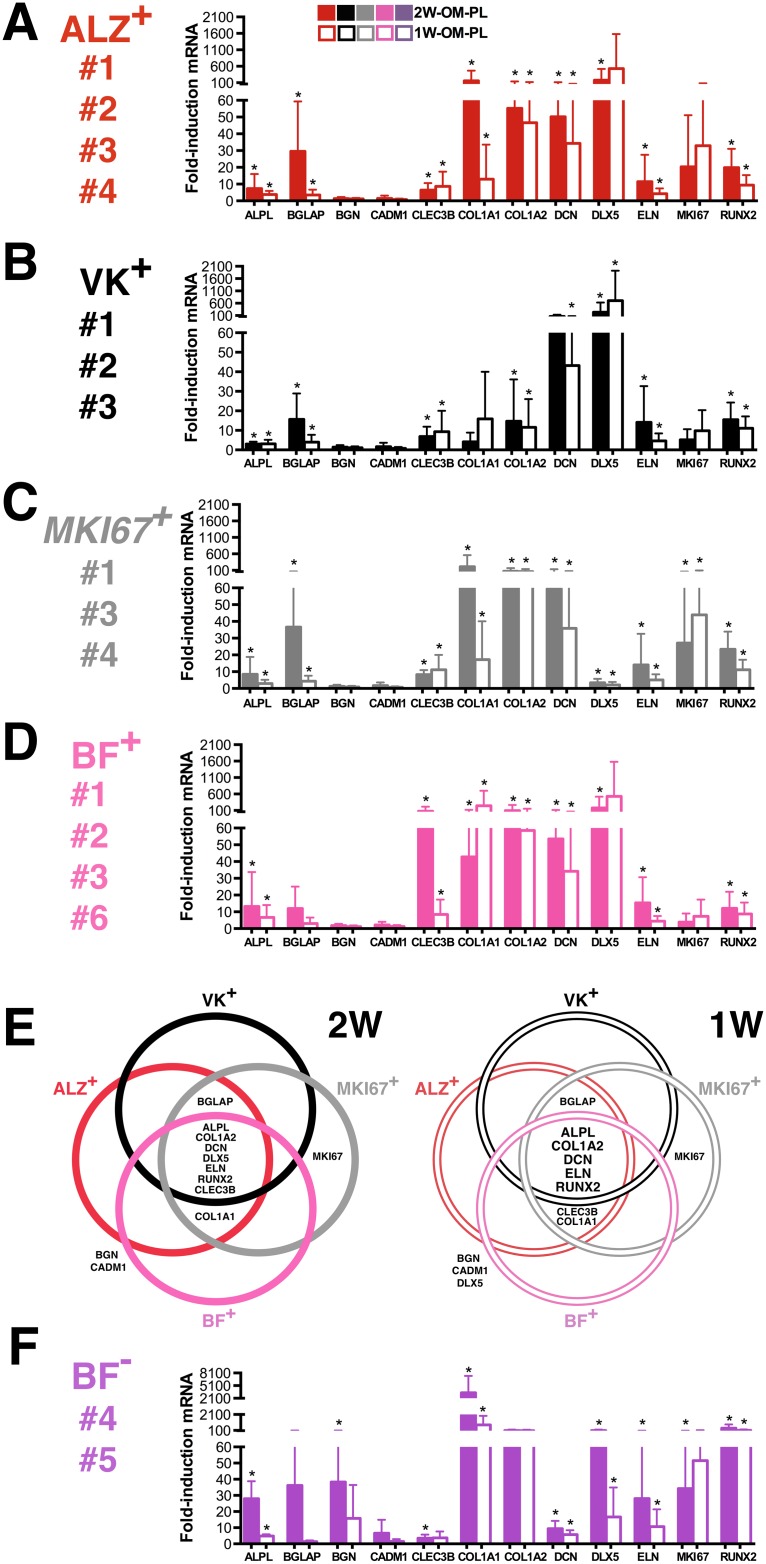
Biomarker characterisation of ALZ^+^, VK^+^, *MKI67*^+^, BF^+^ and BF^-^ cell populations. Histograms of the average extent of osteogenic biomarker gene upregulation in cGMP-hBM-MSC derived from (A) donors #1, #2, #3, #4 with cells positive for Alizarin red S (ALZ^+^) when treated with OM-PL for two weeks (red column) or one week (light column). (B) donors #1, #2, #3 with cells positive for Von Kossa staining (VK^+^) when treated with OM-PL for two weeks (black column) or one week (light column). (C) donors #1, #3, #4 with significant *MKI67*^+^ upregulation when cells were treated with OM-PL for two weeks (grey column) or one week (light column). (D) donors #1, #2, #3, #6 with good bone formation (BF^+^) when cells were treated with OM-PL for two weeks (pink column) or one week (light column). (E) Venn diagrams show the relation between osteogenic function and significantly upregulated biomarkers after (left hand side) OM-PL treatment for two weeks (2W) or (right hand side) OM-PL treatment for one week. (1W). (F) donors #4, #5, incapable of good bone formation (BF^-^) when cells were treated with OM-PL for two weeks (purple column) or one week (light column). Error bars indicate S.D. of means. (*) Constituent mean values were statistically significant (p<0.05).

To accommodate inter-donor heterogeneity, we focused on measuring biomarker expression after OM-PL treatment in phenotypically functional cGMP-hBM-MSC populations. Thus, the representative average biomarker expression associated with the ALZ^+^ phenotype represented donors #1, #2, #3, #4 (Fig 5A); the VK^+^ phenotype represented donors #1, #2, #3 (Fig 5B); the proliferation related *MKI67*^+^ phenotype represented donors #1, #3, #4 (Fig 5C) and the BF^+^ phenotype represented donors #1, #2, #3, #6 (Fig 5D). For each phenotype, a specific subset of the biomarker genes showed statistically significant upregulation among all the relevant donor-specific cGMP-hBM-MSC populations. Statistics for pooled expression patterns for 2W-OM-PL showed seven biomarker genes were significantly upregulated in association with all *ex vivo* osteogenic phenotypes and *in vivo* bone formation (Fig 5E, 2W Venn diagram). However, more relevant to the need for a prompt assay within cGMP cell expansion timelines, after 1W-OM-PL treatment five biomarkers *ALPL*, *COL1A2*, *DCN*, *ELN*, *RUNX2* represented the osteogenic “signature genes” significantly upregulated in all phenotypic contexts (Fig 5E, 1W Venn diagram).

The signature gene induction measurements following OM treatment in the non-bone forming (BF^-^) cGMP-hBM-MSC from donors #4 and #5 (Fig 5F), data in S1 Table, remained statistically significant with one exception. Specifically, *COL1A2* was upregulated ≈177-fold in donor #4 cells yet negligibly (1.1-fold) in cells from donor #5. Another difference was that at two weeks of OM-PL treatment upregulation of *MKI67* was significant for BF^-^ cells (≈13 fold) but negligible for the BF^+^ population, suggesting that cells that did not differentiate had more persistent cell cycle activity. Notably, the two genes constituting the type I collagen protein behaved differently, in contrast to *COL1A2*, *COL1A1* mRNA was not included as a signature gene since it was not significantly upregulated in cells with the VK^+^ phenotype. Of note, *COL1A1* expression at 1W-OM-PL was significantly greater in BF^-^ than in BF^+^ cells (p < 0.001).

### Bioinformatic analysis of signature gene products implicated TGF-ß1 interactions

The Bioinformatic software STRING was used to explore known and predicted interactions for the protein products of the osteogenic biomarker genes. Asking whether the five 1W-OM-PL osteogenic signature genes were preferentially implicated with a particular signaling pathway, we compared their STRING data independently from that of the seven excluded biomarker genes; *BGN*, *BGLAP*, *CADM1*, *CLEC3B*, *COL1A1*, *DLX5*, *MKI67*, whose significant upregulation was only found in a subset of phenotypes.

**Fig 6:**
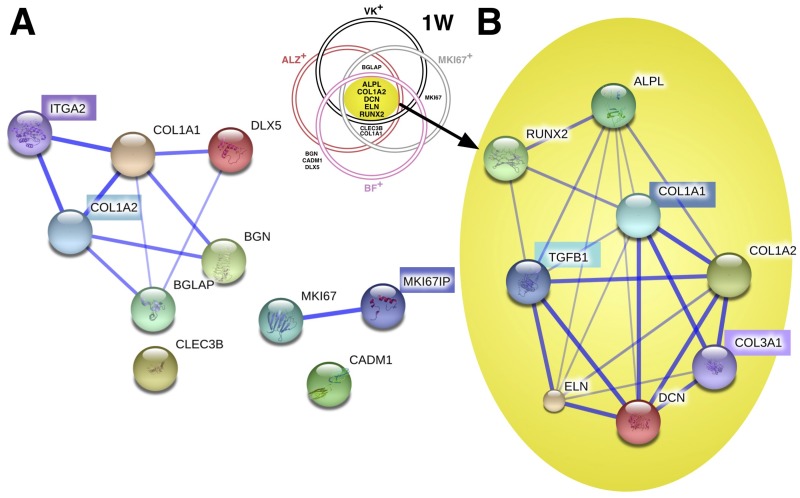
Bioinformatic analysis of osteogenic biomarker gene product interactions. STRING (v9.05) software confidence view, with stronger associations represented by thicker lines, for (A) the osteogenic biomarkers not common to all osteogenic functional phenotypes, constituted a disconnected network. The software predicted three closest functional partners (highlighted acronym) and relative scores were: type I collagen alpha 2 (COL1A2; 0.999); MKI67 interacting nucleolar phosphoprotein (MKI67IP; 0.996) and integrin alpha 2/CD49b; 0.987). (B) the osteogenic signature gene biomarkers common to all osteogenic functional *ex vivo* and *in vivo* phenotypes constituted a closely connected network. The software predicted three closest functional partners (highlighted) and relative scores were: type I collagen alpha 1 (COL1A1; 0.999); Transforming growth factor, beta 1 (TGFB1; 0.999); and type III collagen alpha 1 (COL3A1; 0.993).

For non-signature gene products (Fig 6A), STRING software text-mining evidence indicated associations between COL1A1, DLX5 and BGLAP proteins with co-expression and experimental data also supporting a BGN-COL1A1 interaction, however CADM1, CLEC3B and MKI67 proteins did not show associations. STRING predicted high score functional partners to be COL1A2 (score 0.999), MKI67IP (score 0.996) and ITGA2 (score 0.987). The protein products of two genes CADM1 and CLEC3B remained unassociated.

In contrast, signature gene STRING analysis (Fig 6B) revealed a web of multiple interactions for all five gene-products. Experimental evidence supported interactions between COL1A2, DCN and ELN, all interacting with ALPL that was in turn associated with RUNX2 by text-mining evidence. This implied an interdependent functional network. The three highest score predicted functional partners were COL1A1 (score 0.999), Transforming growth factor beta-1 (TGFß1) (score 0.999) and collagen type III alpha 1 (COL3A1) (score 0.993). STRING experimental evidence supported molecular interactions between ELN, DCN, TGFß1, COL1A1 and COL1A2. Like ALPL and DCN, TGFß1 was associated with six other proteins in the network, highlighting the likelihood of it having a key role in our signature gene network for osteogenesis.

### Osteogenic signature gene expression correlated with bone formation

**Fig 7:**
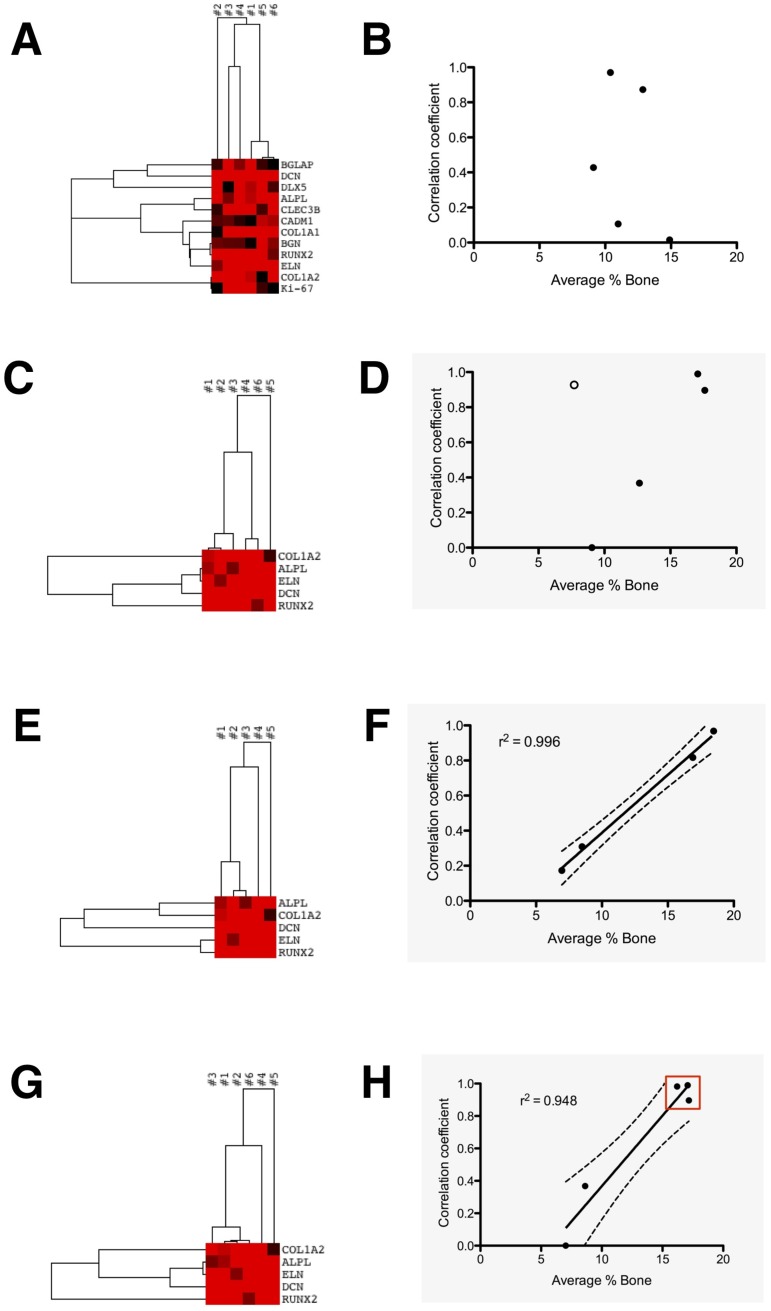
*Ex-vivo* osteogenic signature gene expression could cluster donor-specific cGMP-hBM-MSC populations according to bone forming potential. The gene upregulation profiles of the six donor-specific populations induced for osteogenic differentiation with 1W-OM-PL were subjected to cluster analysis: (A) The dendrogram derived using all twelve osteogenic biomarker genes without prior selection for significance in osteogenic function led to (B) a plot of correlation coefficients between donors and their bone forming potential which revealed no significant relationship (r^2^ = 0.104, p = 0.596). (C) Dendrogram from cluster analysis restricted to the five osteogenic signature genes. Using a Euclidian distance, single linkage algorithm the dendogram indicated closest similarity between bone-forming donors #1 and #2. (D) The plot of correlation coefficients versus bone-forming potential suggested that most associations appeared to constitute a regression slope (closed circles), but there was an outlier association between donors #4 and #6 (open circle) preventing overall correlation (r^2^ = 0.225, p = 0.420). The above cluster analysis was repeated but with modification to accommodate outlying observations. (E) Dendrogram from cluster analysis excluding data from outlying donor #6, restricted to the five osteogenic signature genes, using a Pearson centroid linkage and uncentered correlation distance. (F) The resulting plot of donor correlation coefficients versus bone forming potential confirmed a presumed regression line relationship between bone-forming potential and gene expression for cells from the five congruent donors (r^2^ = 0.996, p = 0.0169). (G) The dendrogram resulting from a Euclidian distance, single linkage algorithm with donor#6 *COL1A2* induction adjusted to 6.63-fold enhanced similarity between bone-forming donors. (H) The corresponding plot of donor correlation coefficients versus bone-forming potential showed a strong linear relationship between *ex vivo* gene expression and *in vivo* bone-forming potential in cells from all donors (r^2^ = 0.948, p = 0.0051) with closely clustered bone-forming donors #1, #2, #3, #6 (red box).

The results supported our initial strategic assumption that correlating *ex vivo* gene expression with *in vivo* bone formation would require identification of a subset of biomarker genes significant for all contexts. Simply using all twelve significantly expressed biomarker genes for agglomerative cluster analysis at 1W-OM-PL (Fig 7A) led to donor correlations unrelated to their respective amount of bone formed *in vivo*, even when using the Pearson centroid linkage that minimized the effect of outlier values, resulting in a very scattered plot (Fig 7B). In contrast, with the five osteogenic signature genes the cluster dendrogram showed more relevant hBM-MSC donor-specific associations (Fig 7C). Close association between bone-forming donors #1 and #2 (correlation coefficient 0.99) led the agglomerative hierarchy. Next, bone-forming hBM-MSC from donor #6 were paradoxically paired with those of non bone-forming donor #4 (correlation coefficient 0.93). Subsequently, hBM-MSC from donor #3 were grouped as similar to the other bone-forming hBM-MSC from donors #1 and #2 (correlation coefficient 0.90). Relatively high correlation coefficients between bone-forming hBM-MSC donors #1, #2, #3 contrasted sharply to the lack of any correlation with donor #5, whose hBM-MSC did not show *ex vivo* osteogenic differentiation or bone formation. Providing a potentially important clue, the dendrogram also highlighted *COL1A2* as the most dissimilarly expressed gene among the six donors. Plotting the dendrogram data (Fig 7D) resulted in slope-aligned donor associations, with a clear outlier exception reflecting the close correlation between donors #4 and #6. Uniquely, these two cell populations both had inconsistent *ex vivo* and *in vivo* phenotypes; the donor #4 hBM-MSCs were positive for *ex vivo* mineralization but did not form bone, however conversely, donor #6 cells had negative mineralization assays yet their xenografts did form bone.

Removing the outlier donor #6 data before centroid-linkage clustering resulted in a dendrogram that prioritised correlations between bone-forming donors (Fig 7E). Plotting the data highlighting a marked linear regression among the remaining five donors (r^2^ = 0.996, p = 0.0022). Thus, bar one exception, the signature gene expression patterns measured in culture allowed discrimination according to the amount of bone formed, placing bone-forming and non-bone-forming hBM-MSC populations at opposite ends of a straight slope (Fig 7F).

Regarding the exceptional association between cells that did not and did form bone, 1W-OM-PL treated donor #4 and #6 cells both showed unusually high *COL1A2* upregulation (152-fold and 199-fold, respectively), in contrast to much more modest *COL1A2* upregulation in the remaining bone forming-donors (#1, 2.63-fold; #2, 3.93-fold; #3, 28.18-fold). Hypothetical mathematical adjustment of donor#6 *COL1A2* upregulation to the central tendency geometric mean value of the other bone-forming donors (6.63-fold) resulted in a new Euclidian distance single linkage cluster dendrogram (Fig 7G). With this mathematical adjustment according to data, the close relationship between osteogenic signature gene expression and bone formation was extended to all six donors (r^2^ = 0.948, p = 0.0051) (Fig 7H), data in S4 Table. Thus, in the context of our signature gene clustering algorithm the elevated *ex vivo COL1A2* expression of BF^+^ donor #6 sufficed to explain its inconsistent outlier status.

### *COL1A2* down-regulation via TGF-ß1 antagonists restored *ex-vivo* matrix mineralization

**Fig 8:**
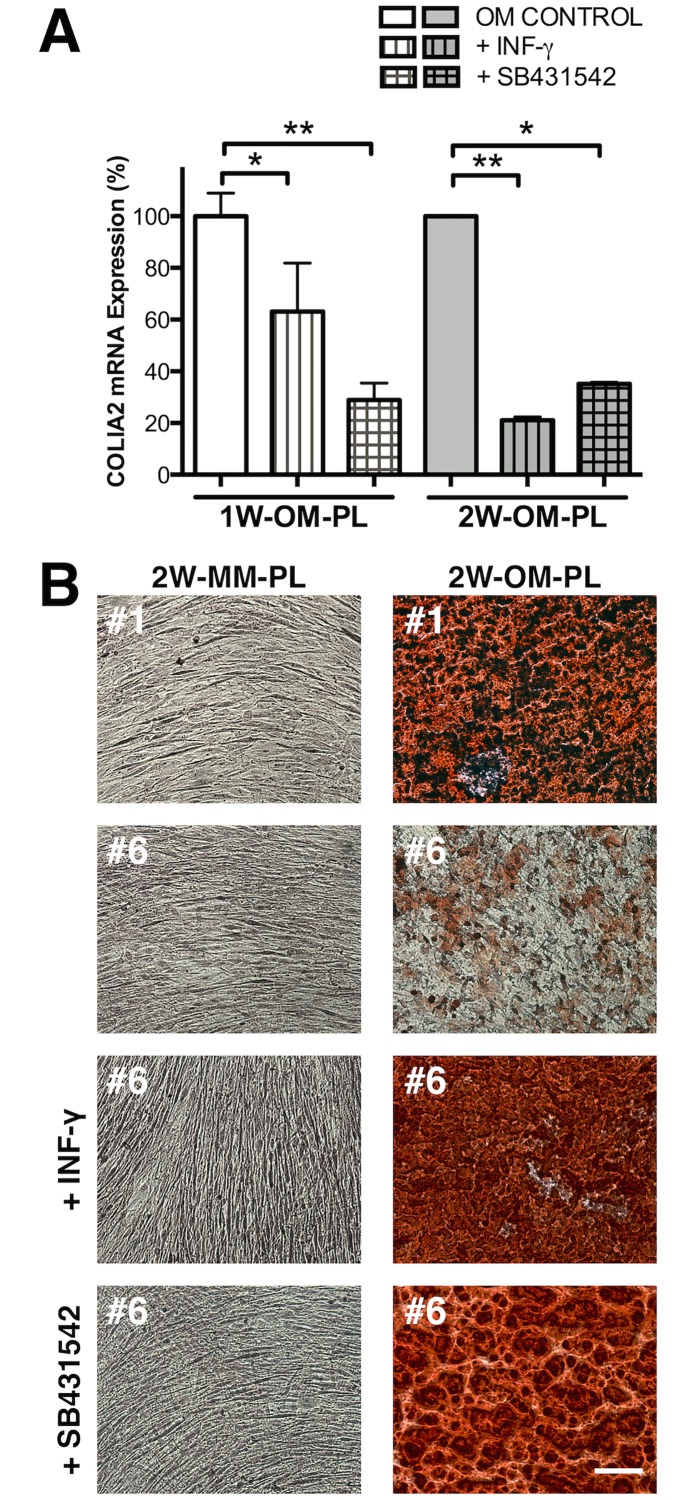
Downregulation of type I collagen restored ex-vivo matrix mineralization. (A) Histogram of RT-PCR determined *COL1A2* mRNA downregulation following treatment of donor #6 cells with Interferon gamma (IFN-γ) or TGF-ß1 signaling inhibitor SB431542 for three days before treatment with osteogenic medium for one or two weeks. *p <0.05, **p <0.005. (B) Representative photomicrographs of Alizarin Red S staining after two-week treatment of cells with maintenance medium (MM) or osteogenic medium (OM) using donor #1 cells as a positive control compared to donor #6 cells and donor #6 cells pre-treated with MM supplemented with 40U/mL INF-γ or 2 μM SB431542 for three days. Bar = 100 μM.

According to our mathematical adjustments, reducing *COL1A2* mRNA levels in donor #6 cGMP-hBM-MSC to 23% of the measured value sufficed to maintain a significant correlation (p≤0.05) between signature gene data and bone formation. We explored whether we could obtain corroborative experimental evidence by a brief three-day treatment of the cells with the physiological factor INF-γ, a cytokine known to antagonise TGF-ß1 mediated induction of collagen expression. Donor #6 cGMP-hBM-MSC treated with either MM alone, MM plus IFN-γ, or MM plus the TGF-beta type I receptor inhibitor SB-431542, grew for three days with similar rates to near-confluence before osteogenic differentiation was initiated with OM-PL. RT-PCR analysis (Fig 8A) showed reduced *COL1A2* induction by 1W-OM-PL after pretreatment with either INF-γ (≈63% of control) or SB431542 (≈29% of control). Moreover these responses were persistent with long-term consequences; the relative level of *COL1A2* induction in donor#6 cells after two weeks of OM-PL treatment remained reduced in cells pre-treated with INF-γ (≈21% of control) or SB431542 (≈35% of control) (Fig 8A), data in S5 Table. With regard to Alizarin Red staining after differentiation, control cells from donor #1 or donor #6 kept in MM-PL, reached a high cell density without mineralization. Following 2W-OM-PL treatment, the positive control donor #1 cells stained strongly with Alizarin Red, but donor #6 cells much less so (Fig 8B). However, donor #6 cells pre-treated with INF-γ or SB431542 for just three days before 2W-OM-PL treatment achieved strong Alizarin red staining.

## Discussion

It is increasingly appreciated that among important rigorous requirements for translation of science into effective therapies, potency assays giving *a priori* indication of administered cell functionality are key for properly conducted ethical trials. Nonetheless, the development of an accurate osteogenic potency assay is particularly challenging. Diverse isolation and culture methods, donor-specific characteristics and methods used to characterise the differentiated phenotype can all contribute to the functional heterogeneity reported for primary cultures of bone marrow derived mesenchymal stem cells. Considering the above we devised a strategy to test whether gene expression of primary cGMP-hBM-MSC *ex vivo* could predict bone forming potential.

The isolation, cell characterisation and culture methods adopted for this study are those currently being employed by the Reborne EU FP7 consortium in phase I clinical trials [40], ensuring that our data should be relevant to clinical contexts. A history of different osteogenic medium (OM) formulations in the literature complicates selection of candidate osteogenic biomarker genes. For example, collagen fibril synthesis and assembly by ascorbic acid-2-phosphate, the long-acting vitamin C derivative is concentration dependent [41]. Beta-glycerophosphate, a substrate for alkaline phosphatase and source of inorganic phosphate is important for the formation of hydroxyapatite Ca_10_(PO_4_)_6_(OH)_2_, yet intracellular incorporation of inorganic phosphate also affects cell function and gene expression [42]. Dexamethasone, a synthetic agonist for the glucocorticoid receptor induces osteogenic differentiation through activation of the Wnt/beta-catenin signaling pathway with variable context and concentration dependent effects on cell proliferation, metabolism and differentiation [43]. We used 10 nM as opposed to 100 nM Dexamethasone to enhance our potential to see osteogenic differences since 100 nM Dexamethasone treatment could artefactually drive matrix mineralization in skin-derived cells [44], co-stimulate expression of adipogenic genes [45] and obscure donor-specific variations in osteoblastic differentiation [46].

Although ascorbic acid, beta-glycerophosphate and dexamethasone sufficed to induce osteogenic differentiation of MSC [47], we added the physiologically relevant supplement BMP-2, that can potentiate differentiation in preosteoblast cells [48, 49]. BMP-2 has a predominant role in the early phase of hMSC differentiation and preserves cell-type specificity since it did not enhance osteogenic differentiation of adipose derived stem cells [50] or periodontal ligament cells [51]. Co-administration of BMP-2 with hMSC increased bone formation in immune deficient mice [52]. It is crucially relevant to graft healing [53] and despite a short half-life [54] BMP-2 is under consideration for clinical applications [55–57]. Our *ex vivo* dose of BMP-2 (100 ng/mL) fell within the range of 25 ng/mL to 25 μg/mL shown to yield near-equivalent amounts of bone *in vivo* [58]. In comparison to some other BMP family members, BMP-2 showed modest improvement in inducing *ex vivo* osteogenic differentiation [59]. Thus BMP-2 was well suited for a discriminatory assay, it wouldn’t necessarily undermine the contribution of a cell’s innate osteogenic potential, as might an alternative excessively dominant inducer of osteogenic differentiation.

The choice of human PL or FBS, both modulators of osteogenic inducers, was considered a predominant characteristic distinguishing OM-PL from OM-FBS. In previous reports, individual hMSC populations had a marginally improved propensity to form bone when grown in PL (9/9; 100%) versus FBS (6/9; 67%), whilst *ex vivo* osteogenic assays were equivalent [60]. Even without osteogenic medium supplements, PL helped prime hMSC towards an osteogenic phenotype by raising levels of *ALPL* mRNA, and matrix proteins integrin-binding sialoprotein IBSP and OPN/SPP1 [61]. The latter secreted protein can bind hydroxyapatite avidly and also act as a cytokine upregulating expression of IFN-γ [62]. Our results supported the view that osteogenic biomarker expression must be considered and interpreted within the context of the specific osteogenic inducers used. For example, serum may be suboptimal for BMP-2 responsiveness [63] and under our conditions *CADM1* expression was not a predictor of hMSC function [64]. Previous predictive biomarkers from model systems using FBS need not necessarily remain relevant when hMSC are derived and cultured with PL. For example, although *ELN* expression may be governed by dexamethasone acting on several glucocorticoid-responsive elements in its promoter [65], its expression was significantly higher in OM-FBS versus OM-PL (p<0–001). This likely reflected complex interactive growth factor networks; e.g. a possible inhibitory effect of basic FGF [66] a significant component of PL [67].

Pooling data from all donors, most genes (7/12; 58%) were significantly induced by either OM-FBS or OM-PL after one week, implying this principally reflected fundamental features of differentiation rather than particular responses to unique growth factors. We confirmed several osteogenic biomarker gene expression patterns reported in an immortalised hMSC-TERT cell model [68] extending their relevance for osteogenic differentiation to primary hMSC with PL-based OM [69]. We also confirmed that early events in the differentiation process were linked to end-stage phenotypic expression such as mineral deposition [70]. Meeting the first of our main aims, prompt differentiation with more robust osteogenic biomarker induction in 1W-OM-PL could facilitate performing the osteogenic potency assay within a cGMP cell expansion time frame.

To categorise heterogeneous donor-specific cultures we focused on significantly expressed genes in cells that functioned positively in multiple osteogenic assays. Alizarin Red and Von Kossa staining for matrix mineralization were complementary staining methods, each reacting with the target calcium molecule differently [32]. Donor #4 cells with their ALZ^+^ yet VK^-^ phenotype highlighted the multifactorial complexity of the matrix deposition and mineralization process. RT-PCR based analysis of *MKI67* expression, a measure for cell cycle activity equivalent to the positive Ki67 index from immunocytochemical staining [71], advantageously provided a phenotypic end-point that could be measured concurrently with the osteogenic biomarker genes at both 1W and 2W time points. In contrast to ALZ^+^ or VK^+^ cells, *MKI67*^+^ cells expressed *BGLAP*, *COL1A1*, *COL1A2* and *RUNX2* at relatively high levels, consistent with observations that collagen type I can enhance hMSC growth rate [72]. hMSC proliferation may be associated open chromatin structures broadening the gene expression repertoire [73, 74] and indicative of multipotency [75] but we could not confirm that proliferation was a reliable correlate with bone formation [25].

The ectopic bone formation assay cleared showed inter-donor heterogeneity and despite a small sample size of six donors, our % incidence of donors with good bone formation (4/6; 67%) was very representative of heterogeneity for successful bone formation in studies with larger sample sizes: (20/34; 59%) [76], (74/120; 62%) [77], (8/14; 57%) [20] and (11/20; 55%) [25]. Parameters such as age of donor, CFU efficiency of primary cultures, or any single gene did not correlate directly with bone formation. Although *BGLAP* was associated with *ex vivo* matrix mineralization it was not strongly associated with the bone forming phenotype, confirming its distinct significance for *ex-vivo* and *in-vivo* osteogenic differentiation pathways [26]. Despite such caveats, seeking promptly expressed osteogenic biomarkers relevant for both *ex vivo* and *in vivo* contexts in 1W-OM-PL data we did identify five genes; *ALPL*, *COL1A2*, *DCN*, *ELN* and *RUNX2* fitting the “signature” of being significantly expressed in all contexts. Providing just five signature genes was advantageous for donor discrimination, since too many clustering variables might reduce the probability of finding clear dissimilarity. Fortunately, only the *RUNX2* and *ELN* genes were co-induced in a parallel manner among the six donors, reducing concern for biased overrepresentation from too many highly correlated variables.

Previous publications aiming to draw correlations between gene expression and bone forming potency have also been based on relatively small sample sizes, yet there is need for caution when interpreting the regression coefficient as an indicator of the relative effectiveness of our signature gene biomarkers. We ensured careful and significant measurement for all our signature genes, because when the variance of individual observations is small, accurate prediction may still be possible despite a small sample size. Given many potential differences between immortalized hMSC-TERT versus primary hMSC expanded and differentiated in different media, the consistent identification of a predictive quality for *COL1A2*, *DCN* and *ELN* expression was striking. Correlation need not imply causation, nonetheless, the five signature genes have well recognised roles in bone formation. Mice null for tissue-nonspecific alkaline phosphatase (*ALPL*) suffer bone abnormalities and its overexpression can increase skeletal mineralization [78]. Mutations in *COL1A2* or its partner *COL1A1* that encode type I collagen can cause dominant inheritance of osteogenesis imperfecta [79]. A strong correlation between the steady-state mRNA levels of type I collagen and periostal bone formation highlights the *in vivo* relevance of its gene expression level [80]. Decorin (*DCN*) a major matrix proteoglycan in bone helps regulate matrix mineralization by influencing collagen assembly [81]. Elastin (*ELN*) degradation products can synergise with TGF-ß1 to promote osteogenic differentiation in fibroblasts [82] and the multifunctional runt related transcription factor 2 (*RUNX2*) is essential for osteoblast development and bone mineralization [83]. It has been suggested that *ex vivo* induction of *ALPL* mRNA levels and ALPL activity could predict the bone forming capacity of human bone marrow stromal cells [84]. We confirmed that testing the response to osteogenic induction can be informative and *ALPL* was one of our five signature genes, however no single genetic biomarker sufficed to reliably predict osteogenic performance among our six donor specific hBM-MSC populations. Rather our data indicated that the measurement of induced levels of expression among a cohort of osteogenic genes was required to reliably correlate *ex vivo* and *in vivo* osteogenesis.

Seeking further clues regarding the role of our five signature genes, a STRING database bioinformatic search identified the three most highly scored associations from known and predicted proteins. As might be expected, COL1A1, the collagen type I triple helix partner of signature gene COL1A2 had the highest interaction score. The STRING database prioritised interactions with morphogenic cytokine TGF-ß1 over the inducing agent BMP-2, a particularly compelling result since counterbalanced BMP-2 and TGF-ß1 signalling pathways converge at the RUNX2 gene to control hMSC differentiation with a coordinated activity that is critical for skeleton formation [85]. TGF-ß1 can enhance BMP-2 ectopic bone formation [86] but in excess can be responsible for inhibition of osteogenesis that can in turn be counteracted with BMP-2 treatment [87]. For the third interactor highlighted by STRING, transgenic mouse studies have confirmed that COL3A1 regulates osteoblastogenesis and the quantity of trabecular bone [88]. The high *in vivo* relevance of these influential signalling pathways [89] may help explain why expression of our osteogenic potency signature genes *ex vivo* could overcome contextual differences to remain relevant for *in vivo* bone formation.

Notably, the most highly upregulated gene for all the osteogenic phenotypes we tested, decorin, was less upregulated in cells from our BF^-^ donors and has been reported to be essential for maintaining mature osteoblasts through an ability to sequester and modulate the activity of TGF-ß1 [90]. A role for decorin in neutralizing the activity of TGF-ß1 would be consistent with observations that neutralizing TGF-ß with specific antibodies could induce an 11% increase in the mineral-to-collagen ratio in murine bone [91] and that excessive TGF-ß1 activity can underlie bone disease [92]. In summary, bioinformatic analysis highlighted that the signature genes form part of a network involving TGF-ß1, which was consistent with this cytokine’s central role in bone remodeling [93].

We also shed light on an apparent anomaly from earlier studies. Despite Collagen type I’s key role in bone formation [94], its *ex vivo* expression has been correlated with bone forming potential [95], but sometimes not [96]. Notably, measurement of baseline *COL1A1* expression was not related to the bone forming potential of hMSC-TERT clones [26], whereas in a follow-up study using the same cells, OM-induced *COL1A2* expression did predict bone formation [27]. The often used, term “collagen type I gene” is ambiguous since two independently regulated *COL1A1* and *COL1A2* genes found on different chromosomes contribute to the two α1(I) chains and one α2(I) chain forming the heterotrimer Type I collagen molecule. These two collagen genes share common [97] and distinct DNA promoter elements [98]. Our data indicated *COL1A2* rather than *COL1A1* gene induction was more precisely correlated with subsequent bone formation. True to the iterative nature of hierarchical cluster analysis, discriminating between the cGMP-hBM-MSC from different donors using the five signature genes required optimization and compensation for apparent outliers. Exclusion of just one outlier, the donor #6 cell population, sufficed to reveal a remarkably linear correlation between the *ex vivo* expression of osteogenic signature genes and bone formation among the remaining five donors (coefficient of determination, r^2^ = 0.996).

Rather than be ignored, the outlier donor #6 was a useful source of hypothesis; since cluster analysis highlighted *COL1A2* as the most disparate signature gene, very highly induced in donor #6 cells. Given that donor #6 cells ultimately did form bone, we conceptualized substituting the donor #6 *COL1A2* high measured value with the lower geometric mean value of the other bone-forming donors. This sufficed to assimilate donor #6 cells with the other bone-forming populations and greatly improve the linear correlation between signature gene expression and bone formation for all donors. The closest clustering of the bone-forming donors with greatest separation from non-bone-forming donors was achieved by the more quantatively discriminatory Euclidian distance and single linkage cluster analysis (r^2^ = 0.948, p = 0.0051). Notably, the phenotype of donor #6 cGMP-hBM-MSC resembled the impaired mineralization and reduced Alizarin red staining in human osteoarthritic (OA) osteoblasts with abnormally high expression of the type I collagen genes [36]. Couchourel et al., rescued Alizarin staining by correcting an abnormal *COL1A1*-to-*COL1A2* expression ratio with inhibition of TGF-ß1 signalling in OA osteoblasts. Given the paradox that donor #6 cells had contrasting *ex vivo* and *in vivo* phenotypes we explored the effects of IFN-γ, a physiologically relevant inhibitor of *COL1A1* and *COL1A2* expression. This cytokine can cross-talk antagonistically with the TGF-ß1 pathway [99] and is produced locally in the bone microenvironment by inflammatory cells as well as hMSC [100] with an anabolic role in bone formation [101]. Brief initial treatment of donor #6 cells with either IFN-γ or an inhibitor of the TGF-ß1 pathway receptor ALK5, significantly reduced subsequent OM induced *COL1A2* gene expression and evoked strong Alizarin red staining. Thus mathematical simulation was qualitatively and quantitatively supported by experimental evidence to confirm that correlations drawn between the levels of signature gene expression and bone formation were consistent with hypothetical and real outcomes.

Additional complementary early parameters of freshly isolated hBM-MSC have recently been proposed for predicting cell growth potential from CFU-F morphology [102] and osteogenic function from monolayer cell morphology [103, 104] or mitochondrial function [105]. However, a key advantage of our RT-PCR based analysis includes the possibility to provide a stored cDNA resource that can be subsequently explored further in relation to patient outcomes and/or as new biomarkers are discovered. Further studies will be needed to help verify our assay and determine whether it may be further improved to meet specific requirements by inclusion of further genes.

In summary, we have provided proof of principal that a promptly performed *ex vivo* potency assay measuring OM induced gene expression of five biomarker “signature genes” could discriminate donor-specific cGMP-hBM-MSC populations according to their bone forming potential *in vivo*. That such linear correlations could be drawn from relatively few samples encourages consideration that the osteogenic potency assay is applicable to individual cases. As cardinal predictive biomarkers, the five signature genes showed very coherent biological qualities consistent with a governing influence of TGF-ß1 signaling in early phases of hMSC osteogenic differentiation. Having demonstrated feasibility, we propose that *ex vivo* functional potency assays will be very valuable for the therapeutic application of adult stem cells.

## Supporting Information

S1 Table qRT-PCR Biomarker mRNA expression in all donor cells. (XLSX)

S2 Table Alizarin Red and Von Kossa Stain quantification. (XLSX)

S3 Table Histological Bone quantification. (XLSX)

S4 Table Hierarchical cluster correlation with bone formation. (XLSX)

S5 Table qRT-PCR COL1A2 mRNA expression in donor#6 cells. (XLSX)
